# Differences in estimates of extinction risk between occupancy and abundance data

**DOI:** 10.1111/cobi.70020

**Published:** 2025-03-28

**Authors:** Mattia Falaschi, Elia Lo Parrino, Raoul Manenti, Gentile Francesco Ficetola

**Affiliations:** ^1^ Department of Environmental Science and Policy Università degli Studi di Milano Milan Italy; ^2^ Laboratoire d’Écologie Alpine Univ. Grenoble Alpes, Univ. Savoie Mont Blanc, CNRS, LECA Grenoble France

**Keywords:** amphibians, demographic decline, IUCN Red List, N mixture models, occupancy models, population trends, Red List assessment, anfibios, declinación demográfica, evaluación de la lista roja, Lista Roja de la UICN, modelo de mezcla *N*, modelo de ocupación, tendencias poblacionales

## Abstract

Temporal trends in populations are often measured with presence–absence and abundance data. These data types are inherently different, but quantitative comparisons of threat statuses assessed through occupancy or abundance data are currently lacking. We applied International Union for Conservation of Nature (IUCN) criteria to estimate extinction risk of amphibians on the basis of data collected over 25 years. We examined whether occupancy and abundance models provided consistent threat status. Occupancy and abundance data suggested declines for the study species in the study area, but occupancy generally showed smaller proportional changes compared with abundance data. Abundance data yielded higher threat categories than occupancy data but were generally associated with larger uncertainties. With abundance data, population declines were found sooner than with occupancy data, but occupancy data estimates were more robust; thus, we advocate the integration of multiple measures of decline when assessing threat status.

## Abstract (es)

Diferencias entre los datos de ocupación y abundancia en las estimaciones de riesgo de extinción

**Resumen:**

Es común que las tendencias temporales de las poblaciones se midan con datos de abundancia y presencia‐ausencia. Estos datos tienen diferencias inherentes, pero actualmente faltan las comparaciones cuantitativas del estado de amenaza por medio de datos de ocupación o abundancia. Aplicamos los criterios de la Unión Internacional para la Conservación de la Naturaleza (UICN) para estimar el riesgo de extinción de los anfibios con base en datos recopilados durante 25 años. Analizamos si los modelos de ocupación y abundancia proporcionaban un estado de amenaza coherente. Los datos de ocupación y abundancia sugirieron declinaciones para el estudio de las especies en el área de estudio, pero la ocupación mostró en general cambios proporcionales menores en comparación a los datos de abundancia. Los datos de abundancia produjeron categorías de amenaza más altas que los datos de ocupación, pero se asociaron casi siempre con una mayor incertidumbre. La declinación poblacional se registró más rápido con los datos de abundancia que con los datos de ocupación, pero las estimaciones de ocupación fueron más robustas, por lo que abogamos por la integración de varias medidas de declinación cuando se evalúe el estado de amenaza.

## INTRODUCTION

Assessing population trends is the first, fundamental step in identifying the underlying drivers of population changes and in determining how to halt and reverse declines. Quantitative measures of species and population trends are also fundamental to identifying species needing conservation and ascertaining extinction risk (Fitzpatrick et al., 2007; Orsenigo et al., 2018). For instance, a decrease in population size is one of the key parameters used by the International Union for Conservation of Nature (IUCN) to identify threatened species and to assign threat categories (IUCN, 2012a).

The data used to estimate temporal trends are usually occupancy or abundance. Abundance provides a direct indication of the number of individuals present in a population; however, accurate abundance measures can be difficult to obtain over large areas, when individuals of a species are hard to detect, or when activity patterns are asynchronous across individuals of the same population (Williams et al., 2002). Occupancy data (i.e., proportion of sites occupied by target species) are often used as an indicator of species abundance instead of number of individuals because a reduction in species occupancy is assumed to be strongly related to a decline in the number of individuals, and presence data are easier to obtain than the number of individuals, thus reducing the burden and cost of monitoring programs (Joseph et al., 2006; Kéry et al., 2010; Steenweg et al., 2018).

**FIGURE 1:**
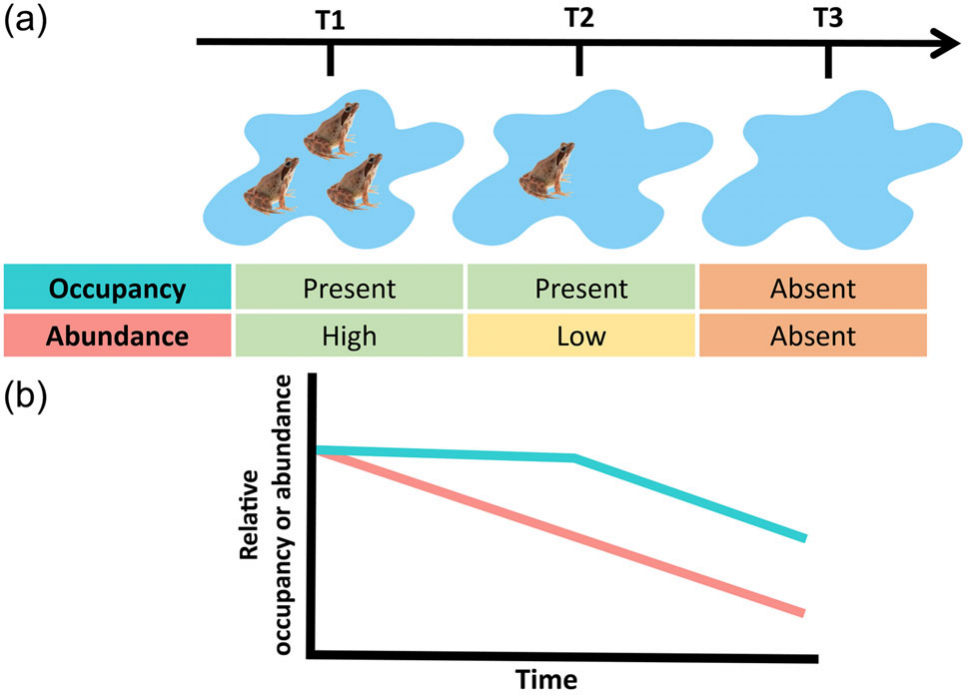
Expected differences in trend estimation from occupancy and abundance data for a hypothetical declining population monitored at 3 different time steps (T1, T2, T3): (a) at T1, the species is present and the population is large; at T2, the population has declined in size, but because the site is still occupied, the occupancy status does not change; and at T3, the population is extirpated; hence, occupancy and abundance are zero. In (b), the scenario described in (a) is extended to multiple sites; occupancy and abundance decline over the entire period, but the relative decline in abundance is larger and is detected earlier compared with occupancy changes.

Abundance and occupancy data have their pros and cons. On the one hand, one expects that declining species will show a reduction in abundance first and later the species’ presence in an area will decline (Figure 1). For this reason, a time lag may occur before population declines are detected with presence–absence data. On the other hand, fluctuations in population size can be large for some animal groups (e.g., insects, amphibians, marine invertebrates), challenging the distinction between natural fluctuations and actual declines (Colebrook, 1985; Pechmann et al., 1991; Wolda, 1992). Therefore, abundance estimates can be more prone to errors than occupancy estimates, and this might amplify the uncertainty of trend assessments (Gutiérrez et al., 2013; Joseph et al., 2006). Additionally, sink populations count as occupied sites, which increases occupancy estimates but has a minor role in species persistence across the landscape (Howe et al., 1991).

When measuring population declines, conservationists often ask 2 related questions. First, is the population stable or is it declining substantially (Q1)? This question can be answered with a *yes* or *n*o or by estimating a probability of decline. Second, what is the actual rate of decline (Q2)? A faster decline signals a higher extinction risk and the need for immediate action (IUCN, 2012a). However, measuring the rate of decline alone is not enough because there is always a level of uncertainty associated with the estimated decline rates.

Both questions are relevant for conservation science, but there are limited quantitative data on the relative effectiveness of abundance versus occupancy to assess population trends and answer these questions. We estimate temporal trends of 4 amphibian species based on occupancy models (detection or no detection data) and abundance models (counts). We then applied IUCN criteria to estimate extinction risk and test whether occupancy and abundance models showed mismatches in threat status through time (IUCN, 2012b). We expect that (Figure 1) abundance would provide earlier warnings of species’ declines; occupancy would show slower rates of decline, and occupancy would provide more precise estimates (i.e., smaller associated errors).

## METHODS

### Study system

From 1996 to 2022, we surveyed 212 amphibian breeding sites in northwestern Italy (Lombardy region) (Appendix S1). Breeding sites encompassed different habitats, including ponds, lakeshores, ditches, and fountains, over a human‐dominated landscape mainly composed of agricultural and urban areas. To assess species trends, each wetland was sampled in 2–9 different years (mean [SD] = 4.4 [1.5]). To maximize the detection of the species and to include detection probability in subsequent models, breeding sites were surveyed multiple times per year, up to a maximum of 8 surveys (mean = 5.1 [1.5] surveys per year).

Monitored sites hosted 10 amphibian species (Falaschi et al., 2021), but we focused on 4 species for which it was possible to produce meaningful estimates of abundance and that were widespread in the study area: agile frog (*Rana dalmatina*), Italian agile frog (*Rana latastei*), Italian crested newt (*Triturus carnifex*), and smooth newt (*Lissotriton vulgaris*). The agile frog and the Italian agile frog are so‐called brown frogs, a group composed of medium‐sized frogs (maximum total length: agile frog 9 cm, Italian agile frog 7.5 cm). Adults are mostly terrestrial and spend only a short reproductive period in an aquatic environment. The agile frog is widespread in southern and central Europe and has relatively broad ecological requirements, whereas the Italian agile frog is endemic to northern Italy and adjacent areas and is a specialist of broadleaved lowland forests (Lanza et al., 2007; Sillero et al., 2014). The 2 newt species often exploit the same breeding and terrestrial habitats but differ considerably in body size (maximum total length: Italian crested newt 15 cm, smooth newt 9 cm). The Italian crested newt occurs only in southern Europe (Italy and some neighboring countries), whereas the smooth newt is much more widespread and is found from western Europe to central Asia (Lanza et al., 2007; Wielstra et al., 2018, 2014). According to the IUCN Red List of Italian vertebrates, the agile frog is classified as least concern, the Italian agile frog is vulnerable, and the 2 newts are near threatened. All the species are considered declining at the national scale (Rondinini et al., 2022).

Field surveys were conducted during the peak of the breeding season of the 4 study species (mainly, February–June). Surveys were performed during the day and at night to optimize detection of different life stages. During each survey, we assessed the presence of amphibian species by visually searching for adults and egg clutches, by listening to the calls of adult males, and by using a dip net to capture tadpoles and larvae (Dodd, 2010; Falaschi et al., 2021). Each nocturnal survey started with a 5‐min call survey to detect the calls of frogs. For all surveys, we visually searched for egg clutches, tadpoles, larvae, and adults over the entire area of the wetland that could be waded. During daytime surveys, in the areas that could be waded, we sampled wetland banks and bottoms with dip nets (Dodd, 2010). The number of observers was usually 2, but varied from 1 to 4. During a survey, each observer walked a section of the wetland; hence, each portion of the wetland was explored only once during a single survey. This allowed us to optimize time when more observers were available and to maintain a stable sampling effort per unit area.

Counts were conducted differently for the frog and newt species. The 2 brown frog species have a short reproductive period that lasts a few days, during which breeding females produce a single egg clutch. Clutches are large and easily identifiable, and clutch counts provide excellent estimates of the actual abundance of breeding females (Campbell Grant et al., 2005; Crouch & Paton, 2000; Falaschi, Gibertini, et al., 2022; Lodé et al., 2005). Therefore, for brown frogs, we carefully inspected the breeding sites and counted the number of clutches for each species during the peak of the breeding period (February and March in the study area). We usually performed one count per season, during the day and at the peak of the breeding period. In cases where we performed multiple clutch counts in the same year, we considered the maximum number of detected clutches for subsequent analyses (Dalpasso et al., 2022; Ficetola et al., 2010).

For both newt species, adults spend longer periods in the aquatic environment, and each female can lay single eggs multiple times, which are small and usually attached to the underside of leaves of aquatic plants (Lanza et al., 2007). Although eggs, larvae, and juveniles can be hard to detect, adult newts are bigger and easier to detect, and the abundance of newts can be estimated based on the number of adults spotted during each survey (Falaschi, Muraro, et al., 2022). Hence, for each nighttime survey, using a torch, we visually searched for adult newts by walking across the entire wadable surface of the wetland to inspect the water column to count adult newts of the 2 species (Falaschi, Muraro, et al., 2022).

### Occupancy models

Imperfect detection can severely bias the estimation of population trends and the assessment of extinction risk (Cruickshank et al., 2016). Therefore, we estimated population trends with occupancy models in a Bayesian framework to jointly model the ecological process and the observational process (Kéry et al., 2013). In the ecological model, we defined the true state *z* of a site *i* at time *t* following a Bernoulli distribution:

(1) $$z_{it}∼\mathrm{Bernoulli}\left(\psi_{it}\right),$$

where ψ is the occupancy probability. Then, to estimate the temporal trend, ψ was related to time:

(2) $$\mathrm{logit}\left[E\left(\psi_{it}\right)\right]∼\alpha +\beta_{\mathrm{trend}}\times \mathrm{yea}r_{t}+T_{t}+S_{i},$$

where α is the intercept (average occupancy); $\beta_{\mathrm{trend}}$ is the temporal trend estimated as the regression coefficient of year against occupancy; $T_{t}$ is a year random effect to account for possible fluctuations of occupancy from the general trend in each year (Kéry & Royle, 2021); and $S_{i}$ is the site random effect, used to account for the nonindependence of the occupancy state at each site in different years.

In the observational model, detection probability was considered by defining the true state *z* following a Bernoulli distribution conditional to the observed state:

(3) $$y_{ijt}|z_{it}∼\mathrm{Bernoulli}\left(z_{it},p_{ijt}\right),$$

where $y_{ijt}$ is the observed state at site *i*, replicate *j*, and year *t*, and *p* is the detection probability of the species. To account for changes in detectability due to the phenology of the species, detection probability was related to date (expressed as day of the year) and hour of the day (expressed as minutes after 06:00) and considered both linear and quadratic effects:

(4) $$\begin{array}{ccc} & & \mathrm{logit}\;\left[E\left(p_{ijt}\right)\right]∼\alpha_{p}+Sp_{i}+\beta_{1}\times \mathrm{dat}e_{ijt}+\beta_{2}\times \mathrm{dat}{e_{ijt}}^{2}\\ & & +\;\beta_{3}\times \mathrm{hou}r_{ijt}+\beta_{4}\times \mathrm{hou}{r_{ijt}}^{2}, \end{array}$$

where $\alpha_{p}$ is the intercept of detection probability, $Sp_{i}$ is a site random effect, and $\beta_{1},\text{…},\beta_{4}$ are the coefficients of the relationship between date, hour, and their quadratic effects with detection probability.

### Abundance models

We implemented 2 different abundance models, one for brown frogs and one for newts. Egg masses of brown frogs are large and easily identifiable, and previous work demonstrated that their detection probability is >90% and that not accounting for imperfect detection is unlikely to bias trend estimates in *R. dalmatina* and *R. latastei* (Falaschi, Gibertini, et al., 2022; Lo Parrino et al., 2024). Hence, the abundance trends of brown frogs were analyzed with a nonhierarchical generalized linear mixed model (Dalpasso et al., 2022). Previous work to estimate the abundance of newts in the same region reported that *N* mixture models used to estimate detection probability based on repeated counts within years fail to converge or returned unrealistic estimates of detectability. Hence, following Falaschi, Muraro, et al. (2022), we used the highest count for each site and sampling season. Still, in *N* mixture models used for the 2 newt species, we retained the detection component and we used the binomial model of Hostetler and Chandler (2015), in which information on detection probability is retrieved from the deviation from the parametric assumptions of population abundance.

In both models, the abundance *N* followed a negative binomial distribution. To estimate temporal trends, *N* was related to time with the following equation, similar to trend estimation with occupancy models:

(5) $$log\left[E\left(N_{it}\right)\right]∼\alpha +\beta_{\mathrm{trend}}\times \mathrm{yea}r_{t}+T_{t}+S_{i},$$

where $N_{it}$ is the abundance in site *i* at time *t*, α is the average abundance, $\beta_{\mathrm{trend}}$ is the temporal trend estimated as the regression coefficient of year against abundance, and $T_{t}$ and $S_{i}$ are random effects of year and site, respectively.

To improve convergence times and avoid an artificial inflation of occupancy and abundance, for each species, we kept only sites within 1500 m of sites that were occupied at least once during the study period. This 1500 m roughly corresponds to the maximum dispersal distance of the 4 study species (Ficetola & de Bernardi, 2004; Jeliazkov et al., 2014; Smith & Green, 2005). Hence, we excluded all areas where each species was absent due to dispersal limitations (Appendix S2). In all models, priors of fixed effects followed a normal distribution with mean = 0 and precision = 0.01. To avoid convergence problems, the prior of $\alpha_{\psi }$ was defined as $\mathrm{logit}(\alpha_{\psi })$ following a uniform distribution bounded between 0 and 1. Prior of $\alpha_{N}$ followed a uniform distribution bounded between −10 and 10. Priors of the year and site random effects followed a normal distribution with mean = 0 and standard deviation following a uniform distribution bounded by 0 and 10. All models were implemented in nimble, which allows writing custom Bayesian models in the bugs language (de Valpine et al., 2017). Models were run with 3 Markov Chain Monte Carlo for 400,000 iterations. The first 300,000 iterations were discarded as burn‐in, and we thinned the sample to obtain 1000 posteriors for each chain. Before running the models, the continuous year variable was centered so that the year 2009 corresponded to zero. The convergence of the models was evaluated by visual inspection of the chains and by looking at Rhat values. The scripts of models and data used to run the analyses are available from figshare (https://doi.org/10.6084/m9.figshare.23765361.v2).

### Comparing occupancy and abundance trends

Trends were compared through the use of derived parameters, which are parameters derived from calculations made at each iteration of the model and enable the propagation of parameter uncertainty (Kéry, 2010). To compare trend estimates of occupancy and abundance over the whole study period, we calculated a derived parameter to indicate the proportional change of occupancy and abundance over the study period:

(6) $$\mathrm{Proportional}\;\mathrm{change}=\frac{\mathrm{po}p_{t_{f}}}{\mathrm{po}p_{t_{0}}},$$

where $\mathrm{po}p_{t_{f}}$ and $\mathrm{po}p_{t_{0}}$ are, respectively, the estimated occupancy and abundance of populations of target species in the last and first years, based on the population trend (i.e., excluding random yearly fluctuations). For instance, $\mathrm{po}p_{t_{f}}$ for occupancy was a derived parameter:

(7) $$\mathrm{po}p_{t_{f}}=\sum_{i=1}^{n}\mathrm{logistic}\left(\alpha +\beta_{\mathrm{trend}}\times \mathrm{yea}r_{t_{f}}+S_{i}\right),$$

where $t_{f}$ is the last year of sampling, *n* is the number of sites, and α, $\beta_{\mathrm{trend}}$, and $S_{i}$ are parameters estimated in abundance and occupancy models (intercept, regression coefficient between year and occupancy and abundance, and site random effect).

### Estimation of extinction risk

To assess whether different data types (occupancy or abundance) deliver different conclusions about conservation status of species at different time steps, we estimated the extinction risk following the A2 criterion of the IUCN (IUCN Standards and Petitions Committee, 2022). We assumed population decline had not ceased and classified species as least concern (LC) for declines <20%, as near threatened (NT) for declines ≥20%, as vulnerable (VU) for declines ≥30%, as endangered (EN) for declines ≥50%, and as critically endangered (CR) for declines ≥80% over 10 years or 3 generations, whichever was longer (IUCN, 2012a).

We divided the time frame of the study into 3 periods. Each period contained a similar number of sampling years (period 1: 1996–2003 [τ_1_]; period 2: 2004–2011 [τ_2_]; period 3: 2017–2022 [τ_3_]; no surveys were conducted from 2012 to 2016). Then, for each species and period, we calculated the mean occupancy and abundance across the years included in a given period. Mean occupancy in a period ($\psi_{\tau }$) was calculated by averaging the estimated number of occupied sites across the years included in each period. Mean abundance in a period ($N_{\tau }$) was calculated by averaging the estimated total abundance across all sites across the years included in each period (details in Appendix S3). Average occupancy and abundance of each period were calculated as derived parameters (for each posterior) to keep the uncertainty of these estimates.

Average occupancy and abundance were then used to assess extinction risk following the IUCN criteria over 2 time steps (τ_1_ − τ_2_ and τ_2_ − τ_3_). For each species, we calculated the decline of occupancy $D_{\psi }$ and abundance $D_{N}$ as

(8) $$D_{\psi }=1-\frac{\psi_{\tau }}{\psi_{\tau -1}},$$

(9) $$D_{N}=1-\frac{N_{\tau }}{N_{\tau -1}},$$

where τ indicates occupancy or abundance in periods τ_2_ and τ_3_, and τ−1 is the occupancy/abundance in each respective previous period (τ_1_ and τ_2_). Starting from these derived parameters, we calculated 2 indicators to answer Q1 and Q2: first, decline probability ($D_{p}$), defined as the percentage of posteriors <0 when calculating $D_{\psi }$ and $D_{N}$, and second, decline rate ($D_{r}$). Decline rate was calculated as follows: declines between periods ($D_{\psi }$ and $D_{N}$) were transformed to annual declines ($D_{a}$) with the formula

(10) $$D_{a}=1-{\left(1-D\right)}^{\frac{1}{L}},$$

where *D* is the total decline of occupancy or abundance between 2 consecutive periods ($D_{\psi }$ or $D_{N}$) and *L* is the elapsed time. In our case, *L* was 8 years between the first (τ_1_) and second (τ_2_) periods and 12 years between the second (τ_2_) and third (τ_3_) periods. Subsequently, annual declines were transformed into decline rates ($D_{r}$) with

(11) $$D_{r}=1-{\left(1-D_{a}\right)}^{\mathrm{GL}},$$

where $D_{a}$ is the annual decline calculated in the previous step and GL is a species‐specific constant representing the longest period between 10 years or 3 times the generation length (Bird et al., 2012; Tracewski et al., 2016) (Appendix S4).

## RESULTS

**FIGURE 2:**
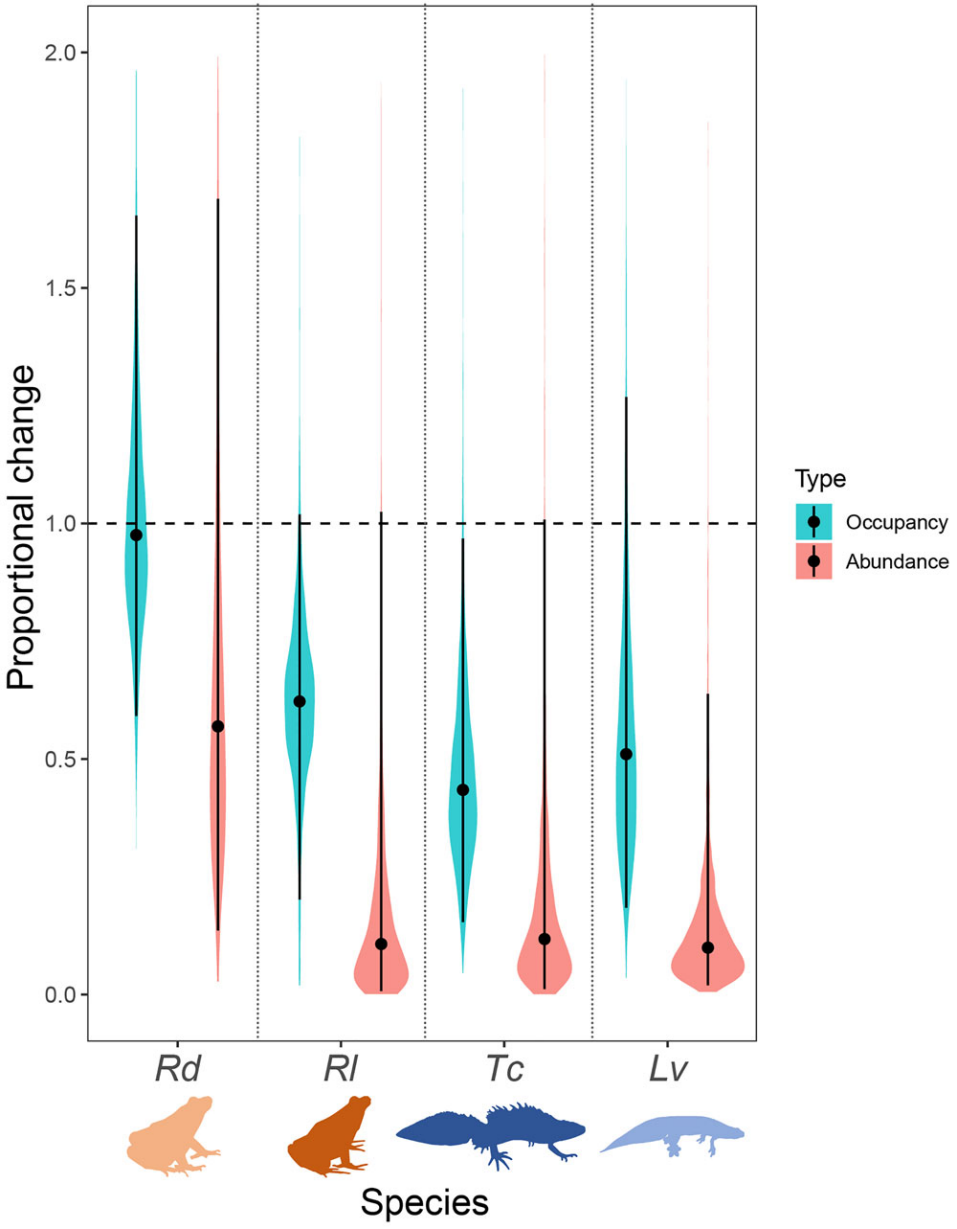
Posterior distribution of the relative changes (expressed as proportions) in total occupancy and abundance across all study sites for the entire study period (1996–2022) in 4 amphibian species (shading, entire posterior distributions; dots, median; whiskers, 95% credible intervals; horizontal dashed line, threshold of 1 [no overall relative change]; *Rd*, *Rana dalmatina*; *Rl*, *Rana latastei*; *Tc*, *Triturus carnifex*; *Lv*, *Lissotriton vulgaris*).

All occupancy models reached convergence, with all parameters showing Rhat values <1.1 (Appendix S5). For the abundance models, most parameters reached very good convergence, but, in a few cases, yearly estimates of total abundance showed Rhat values slightly >1.1 (1 parameter for smooth newt, 2 for Italian crested newt and agile frog, 4 for Italian agile frog) (Appendix S5), indicating more uncertainty and need of large sampling effort for accurate estimation of abundance. Still, convergence was successfully attained for the key estimated parameters (estimates of trend and proportional change). For almost all species, average changes in occupancy and abundance were negative, indicating a general decline. The exception was the occupancy of *R. dalmatina* (Appendix S6). Alternative models for the 2 newt species without the detection component showed strongly consistent results (Appendix S7). Therefore, we present the models including the detection component that more realistically represents the count error associated with field surveys. When we considered the whole study period, the 95% credible intervals (CIs) for the proportional change parameter (Equation 6) did not overlap 1 (i.e., the value indicating a stable population trend) for *T. carnifex* in occupancy models and for *L. vulgaris* in abundance models (Figure 2). Still, the generalized decline was evident for 3 out of 4 species (Appendix S5; Table 1).

**TABLE 1:** Probabilities of decline^a^ and decline rates^b^ of 4 amphibians estimated by occupancy and abundance models over 2 periods (*τ*_1_ − τ_2_, 8 years; τ_2_ − *τ*_3_, 12 years).

		Occupancy			Abundance		
		Decline probability	Decline rate		Decline probability	Decline rate	
Species	Period^c^		Median	95% CI		Median	95% CI
*Rana dalmatina*	τ_1_ − τ_2_	0.42	+0.03	−0.23 to 0.53	0.76	−0.34	−0.92 to 0.93
	τ_2_ − τ_3_	0.64	−0.03	−0.14 to 0.15	0.91	−0.29	−0.58 to 0.16
*Rana latastei*	τ_1_ − τ_2_	0.80	−0.09	−0.30 to 0.19	0.87	−0.71	−0.98 to 1.87
	τ_2_ − τ_3_	0.99	−0.20	−0.31 to −0.06	0.97	−0.63	−0.92 to 0.04
*Triturus carnifex*	τ_1_ − τ_2_	0.98	−0.35	−0.61 to 0.01	0.97	−0.92	−0.99 to 0.28
	τ_2_ − τ_3_	0.97	−0.29	−0.49 to −0.02	0.75	−0.36	−0.92 to 1.13
*Lissotriton vulgaris*	τ_1_ − τ_2_	0.88	−0.22	−0.48 to 0.19	0.99	−0.77	−0.95 to −0.25
	τ_2_ − τ_3_	0.96	−0.23	−0.44 to −0.02	0.96	−0.45	−0.75 to 0.08

^a^ Proportion of negative posteriors when calculating proportional population changes between the 2 periods ($D_{p}$).

^b^ Follows A2 criterion of the International Union for Conservation of Nature (see $D_{r}$ in “METHODS”).

^c^ Periods: τ_1_ − τ_2_, decline probabilities 1996–2003 and 2004–2011; τ_2_ − τ_3_, decline rates 2004–2011 and 2017–2022.

**FIGURE 3:**
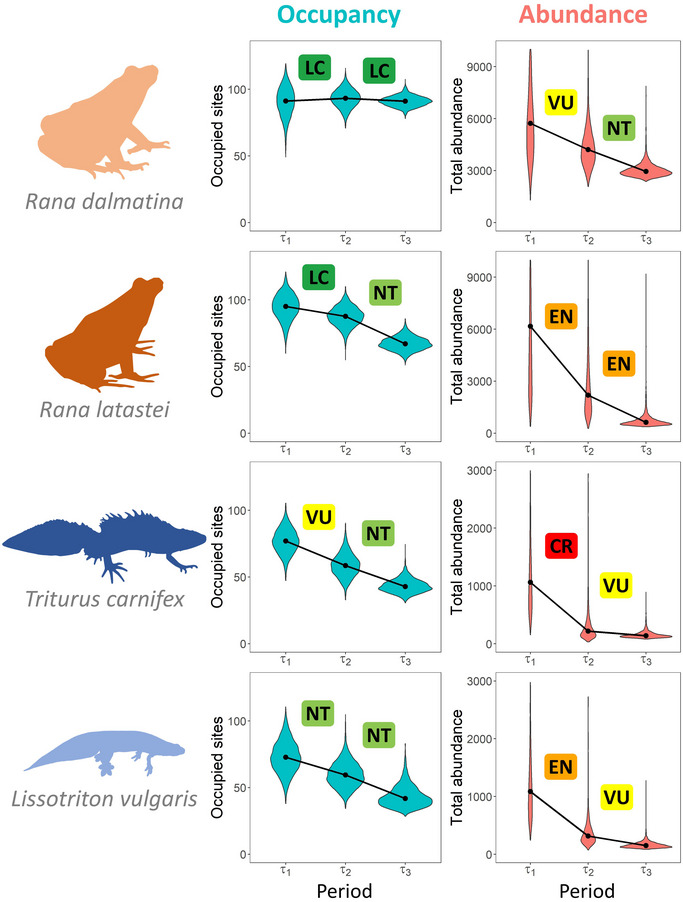
Number of occupied sites and total abundance across all study sites for the 4 amphibian species in 3 periods over 27 years (colored rectangles, extinction risks based on population changes between a period and the previous one; LC, least concern; NT, near threatened; VU, vulnerable; EN, endangered; CR, critically endangered; graph elements as in Figure 2).

Larger uncertainties were generally associated with changes in abundance for analyses over the 3 periods (Figure 3). Decline probabilities from τ_1_ to τ_2_ (first time step) were generally lower than those from τ_2_ to τ_3_ (second time step) (Table 1). For instance, the decline probability of occupancy for *L. vulgaris* was 0.88 over the first time step and 0.96 over the second step. An exception was the abundance of *T. carnifex*, which showed a decline probability of 0.97 over the first time step and 0.75 over the second step (Table 1).

Decline rates obtained through occupancy and abundance were often not consistent (Table 1). For occupancy, declines were generally weak for brown frogs, almost unchanged for *R. dalmatina*, and weak for *R. latastei* in both periods (Table 1). The 2 newts showed larger declines of occupancy, between 20% and 30% in both periods (Table 1). Abundance had a different pattern. Declines were steep between τ_1_ and τ_2_ and less steep between τ_2_ and τ_3_ (Table 1; Figure 3). The rates of decline estimated by occupancy models always placed both frog species in low‐rank threat categories (between least concern and near threatened), whereas newts were placed in threat categories ranging from near threatened to vulnerable (Figure 3). Despite large uncertainties, abundance data returned extinction risk always 1 or 2 (in one case even 3) IUCN Red List categories higher than occupancy, with the largest differences at the beginning of the study, when the largest declines in abundance occurred (Figure 3; Table 1). During the first period, the extinction risk based on abundance data ranged between vulnerable (*R. dalmatina*) and critically endangered (*T. carnifex*).

## DISCUSSION

Our analyses of population trends showed substantial differences in extinction risks estimated from occupancy or abundance data. Abundance models showed larger uncertainties but generally suggested higher threat categories. Declines were detected earlier with abundance models than with occupancy models, suggesting that abundance data might be better for rapidly tracking species trends (Figure 3). The rapid implementation of conservation actions is crucial to prevent species extinctions, given that delays can often doom conservation plans to failure (Hammer et al., 2015; Martin et al., 2012). Prompt detection of declines is important so that conservation actions can immediately target shrinking populations. In our case study, relying only on occupancy data would have led to the conclusion that just one species (*T. carnifex*) was undergoing substantial declines in the first years of monitoring. Still, abundance data revealed that the strongest declines took place at the beginning of the study period, where all species faced declines of 34–92% on average (Table 1; Figure 3).

The apparent ability of abundance models to detect population declines earlier does not come without drawbacks. First, the collection of reliable abundance data is usually more effort demanding because individual detection probabilities are lower than species detection probabilities. This implies that more surveys are generally needed to assess true abundance with sufficient precision (Ficetola et al., 2018; Joseph et al., 2006). The IUCN Red List assessments should always deal with uncertainty (IUCN Standards and Petitions Committee, 2022); therefore, comparing extinction risks among species might be more complex, with the larger uncertainty associated with abundance data. In some cases, large uncertainty may reflect the natural fluctuations of some species or populations, which can make the use of abundance data inappropriate for assessing threat status (IUCN Standards and Petitions Committee, 2022).

Retrieving abundance data may require species‐specific protocols. For instance, in our case study, we used visual counts of different life stages for different species, counting egg clutches for frogs and adult individuals for newts. Searching and counting all the individuals generally requires much more time than just detecting a few of them. Retrieving abundance data for multiple species simultaneously or for the whole community can thus become resource intensive, and this may reduce the number of sites surveyed per time unit and increase the cost of monitoring programs (Devarajan et al., 2020; Southwell et al., 2019). Furthermore, in some cases, occupancy analyses might be a better a priori choice for taxa for which the definition of an individual is not straightforward (e.g., some plants and lichens) or when abundance is not strongly related to the persistence of the species over a large scale (e.g., in sink populations attracting large numbers of individuals [Hanski et al., 1995; Thomas et al., 1999]).

Although we evaluated threat status based on IUCN Criterion A (population size reduction, which can be assessed through multiple indicators, including number of individuals and number of populations), species assessments should consider all the possible criteria (IUCN, 2012a). Therefore, the rates of decline estimated with different approaches (Table 1) should be considered jointly with additional parameters that are relevant to the other criteria (e.g., B, range size; C, total population size).

Although assessing threat status and setting conservation priorities are 2 distinct processes (Collen et al., 2016), knowing the conservation status of a species is a first and crucial step for subsequent conservation prioritizations (Fitzpatrick et al., 2007). In this context, prioritizing conservation actions based solely on occupancy changes can underestimate the actual extinction risk and thus impair conservation prioritizations (Figure 3). However, acknowledging the limitations of occupancy‐derived trends can be complex when the presence or absence of a species is the only information available and abundance data are lacking. Although several researches found positive correlations between abundance and occupancy, the shape and strength of this relationship are often species specific and possibly location specific (Gaston et al., 2000; Steenweg et al., 2018; Ten Caten et al., 2022; Verberk et al., 2010). This can hamper efforts to overcome the problem of underestimating threat status based on occupancy data.

In the absence of a benchmark to correct for the occupancy–abundance mismatch, some measures can be applied at the policy level. For instance, looking at occupancy data only, our models suggested large declines in newts (e.g., *T. carnifex* immediately spotted as VU [Figure 3]), whereas *R. dalmatina* did not emerge as threatened. Previous analyses showed that both species are negatively related to the occurrence of invasive predators (Bélouard et al., 2019; Falaschi et al., 2021; Vannini et al., 2018); therefore, conservation actions aimed at restoring or creating predator‐free wetlands could benefit both newts and frogs (Rowe & Garcia, 2014). Because of the occupancy–abundance mismatch, prioritizing actions that are expected to benefit most species, even the ones not apparently declining, might be the optimal strategy to allocate conservation efforts. We simplified the application of IUCN criteria because we applied them directly to the study populations without assuming potential connections with conspecific populations outside the study area. An accurate regional assessment of threat level should consider the possibility that immigration from nearby regions would reduce extinction risk (IUCN, 2012b). The application of estimates of decline based on the assumption that nearby populations are connected may deliver different results, possibly reducing the differences in threat status between abundance and occupancy data. The threat status may be different at a larger spatial scale (Hecnar & M'Closkey, 1996, 1997). Nevertheless, the high levels of urbanization and fragmentation in the study area mean that amphibian populations are extremely isolated; thus, it is unlikely that immigration will effectively rescue populations (Bani et al., 2015; Ficetola et al., 2007).

We calculated threat status based on occupancy or abundance in the previous period; hence, we performed 2 independent assessments. Our analyses showed that species showing a steady decline were placed twice in the same category (e.g., *L. vulgaris*, which was placed twice in the NT category [Figure 3]). This problem is not considered in the IUCN guidelines, for which relatively short time intervals (10 years or 3 generations) are generally considered. Nevertheless, when available, information on long‐term trends can be extremely relevant when setting priorities for conservation. For instance, the repeated placement of a species in a high‐threat category could be considered an indication that the species is not responding to ongoing conservation actions and could trigger the development of new efforts. An even more striking pattern of mismatch between IUCN Red List category and the actual population status was evident from the abundance of *T. carnifex* (Figure 3). In our exercise, the category of this species improved from CR to VU, but total abundance continued to declined from the first to the second assessment (Figure 3; Appendix S6). This means that a species status can improve even if the population is declining, which can be a complex issue to communicate to policy makers and to a broad audience. Given the central role of the IUCN in guiding conservation priorities, funding allocation, and policy development (Betts et al., 2020), downlisting a species that is declining due to a decreased rate of population reduction might be problematic. This issue may be particularly relevant in the next few years, given the growing number of taxa that are being reassessed by the IUCN.

**FIGURE 4:**
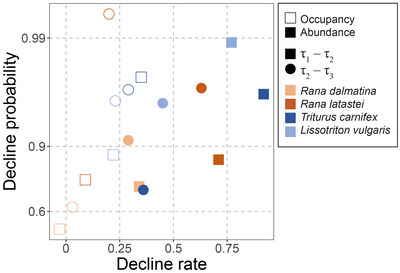
Decline rates ($D_{p}$) and decline probabilities ($D_{p}$) from occupancy and abundance models of 4 amphibian species calculated over 2 time steps (open shapes, estimates of occupancy models; solid shapes, estimates of abundance models; squares, changes over the first time step [between period τ_1_ and τ_2_]; circles, changes over the second time step [between period τ_2_ and τ_3_]; decline probability, range 0–1, was transformed to better show differences among the highest probability values with the formula $-\log[1-D_{p}]$).

Population trends estimated from occupancy and abundance data can differ substantially in the magnitude and in the timing of changes, and species showing stable occupancy trends can actually be numerically decreasing at rates that would indicate a threatened status (Figure 3). These differences challenge the comparison of threat status derived from different data across a wide range of species, which is one of the main goals of IUCN Red List. Certainly, calling for an increase in sampling to preferentially collect abundance data could partially overcome these issues, but it might not be feasible for all species. The optimization of statistical models integrating occupancy and abundance to infer abundance declines based on occupancy data might be another option (Dorazio, 2007), but the feasibility and reliability of these approaches and the time required to develop them are currently unknown (Freckleton et al., 2005; Steen et al., 2023; Zuckerberg et al., 2009). In this context, integrating more parameters into the assessment of conservation status can benefit the meaningfulness of subsequent conservation policies. For instance, the choice of the threat status and prioritization of conservation efforts could be based on both decline rate and decline probability (Figure 4). This strategy would allow one to distinguish between species with strong or weak declines and large associated uncertainties and species for which the certainty of population trend is high.

## AUTHOR CONTRIBUTIONS

Mattia Falaschi and Gentile Francesco Ficetola designed the study. Mattia Falaschi performed the analyses. All authors participated in data collection and maintenance and contributed substantially to the writing of the manuscript.

## Supporting information



Appendix S1. Study area and amphibian breeding sites monitored in Northern Italy.
Appendix S2. Selection of species‐specific study sites.
Appendix S3. Calculation of the mean occupancy and abundance across three discrete periods.
Appendix S4. Correction factors used to transform declines among periods to annual declines.
Appendix S5. Parameters estimated by occupancy and abundance models for the four study species.
Appendix S6. Species‐specific trends of occupancy and abundance.
Appendix S7. Comparison of abundance models for *Triturus carnifex* and *Lissotriton vulgaris*, with or without including a detection component in the models.
