# Supplementary material for: Differences in estimates of extinction risk between occupancy and abundance data
Source: Conserv Biol. 2025 Mar 28;39(4):e70020. doi: 10.1111/cobi.70020 (PMC12309650; doi:10.1111/cobi.70020)
Supplement: Supplementary file 1 — Appendix S1. Study area and amphibian breeding sites monitored in Northern Italy. Appendix S2. Selection of species‐specific study sites. Appendix S3. Calculation of the mean occupancy and abundance across three discrete periods. Appendix S4. Correction factors used to transform declines among periods to annual declines. Appendix S5. Parameters estimated by occupancy and abundance models for the four study species. Appendix S6. Species‐specific trends of occupancy and abundance. Appendix S7. Comparison of abundance models for Triturus carnifex and Lissotriton vulgaris, with or without including a detection component in the models. [file COBI-39-e70020-s001.pdf]

Supporting Information for:

Differences in estimates of extinction risk between occupancy and abundance data

Table of contents:

- **Appendix S1.** Study area and amphibian breeding sites monitored in Northern Italy.
- **Appendix S2.** Selection of species-specific study sites.
- **Appendix S3.** Calculation of the mean occupancy and abundance across three discrete periods.
- **Appendix S4.** Correction factors used to transform declines among periods to annual declines.
- **Appendix S5.** Parameters estimated by occupancy and abundance models for the four study species.
- **Appendix S6.** Species-specific trends of occupancy and abundance.
- **Appendix S7.** Comparison of abundance models for *Triturus carnifex* and *Lissotriton vulgaris*, with or without including a detection component in the models.

Appendix S1. Study area and amphibian breeding sites monitored in Northern Italy. Orange circles represent study sites, and background colours represent the land cover, retrieved from a land cover map of the Lombardy region (https://www.geoportale.regione.lombardia.it/; ground resolution 3 m). The location of the study area within Italy is highlighted in red in the top right inset.

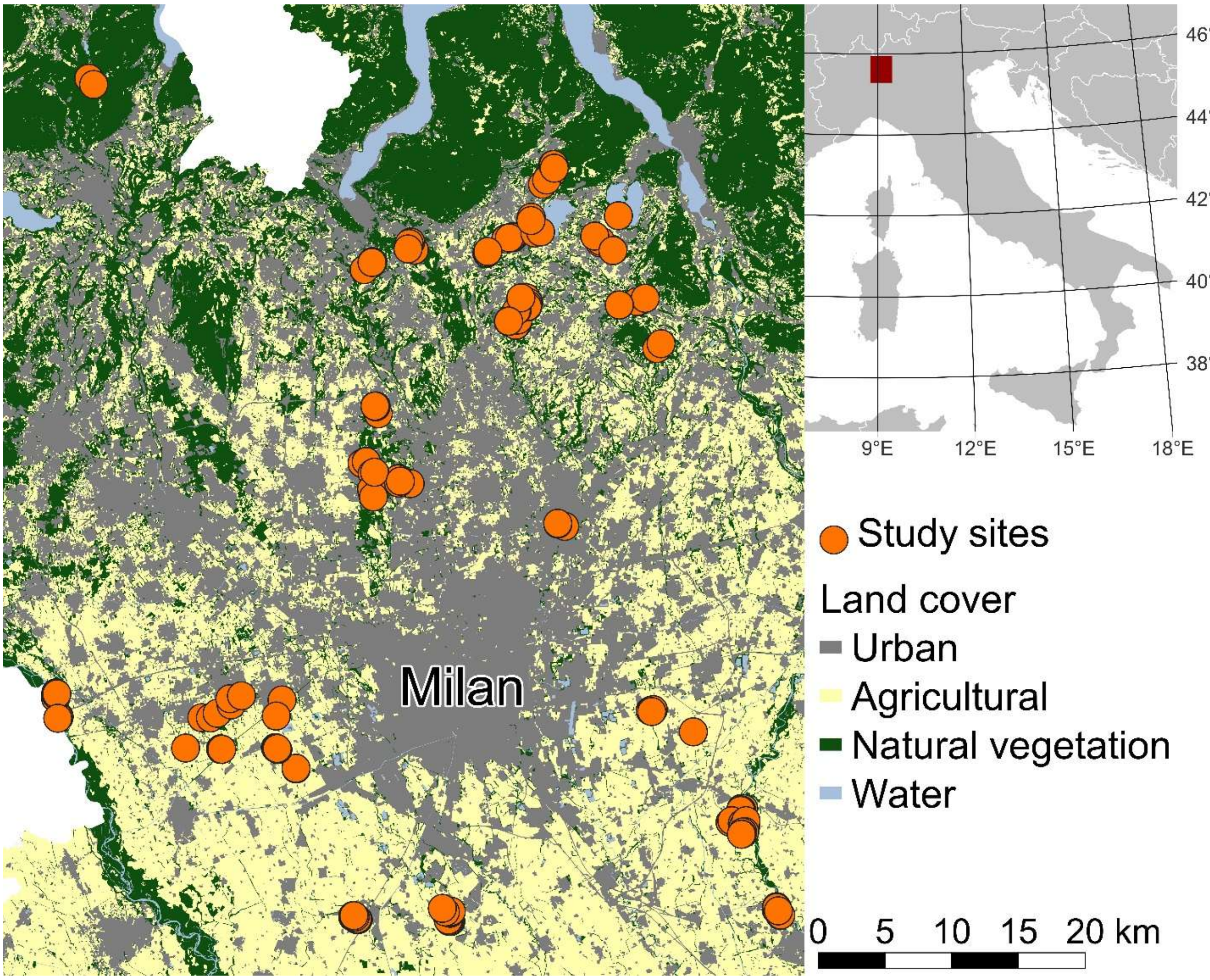

Appendix S2. Selection of species-specific study sites.

Including sites that cannot be reached due to dispersal limitations can bias results of species distribution models (Godsoe 2010). Therefore, we excluded sites where the focal species was never detected throughout the whole sampling period and that were too isolated to be reached by dispersing individuals. For each species we kept only sites within 1,500 m from sites where the species were found at least once during the study period. 1,500 meters roughly correspond to the maximum dispersal distance of the four study species (Ficetola & De Bernardi 2004; Smith & Green 2005; Jeliazkov et al. 2014). This procedure resulted in four set of breeding sites, one for each species, including 126 sites for *Rana dalmatina* (a), 127 for *Rana latastei* (b), 111 for *Triturus carnifex* (c), and 131 for *Lissotriton vulgaris* (d).

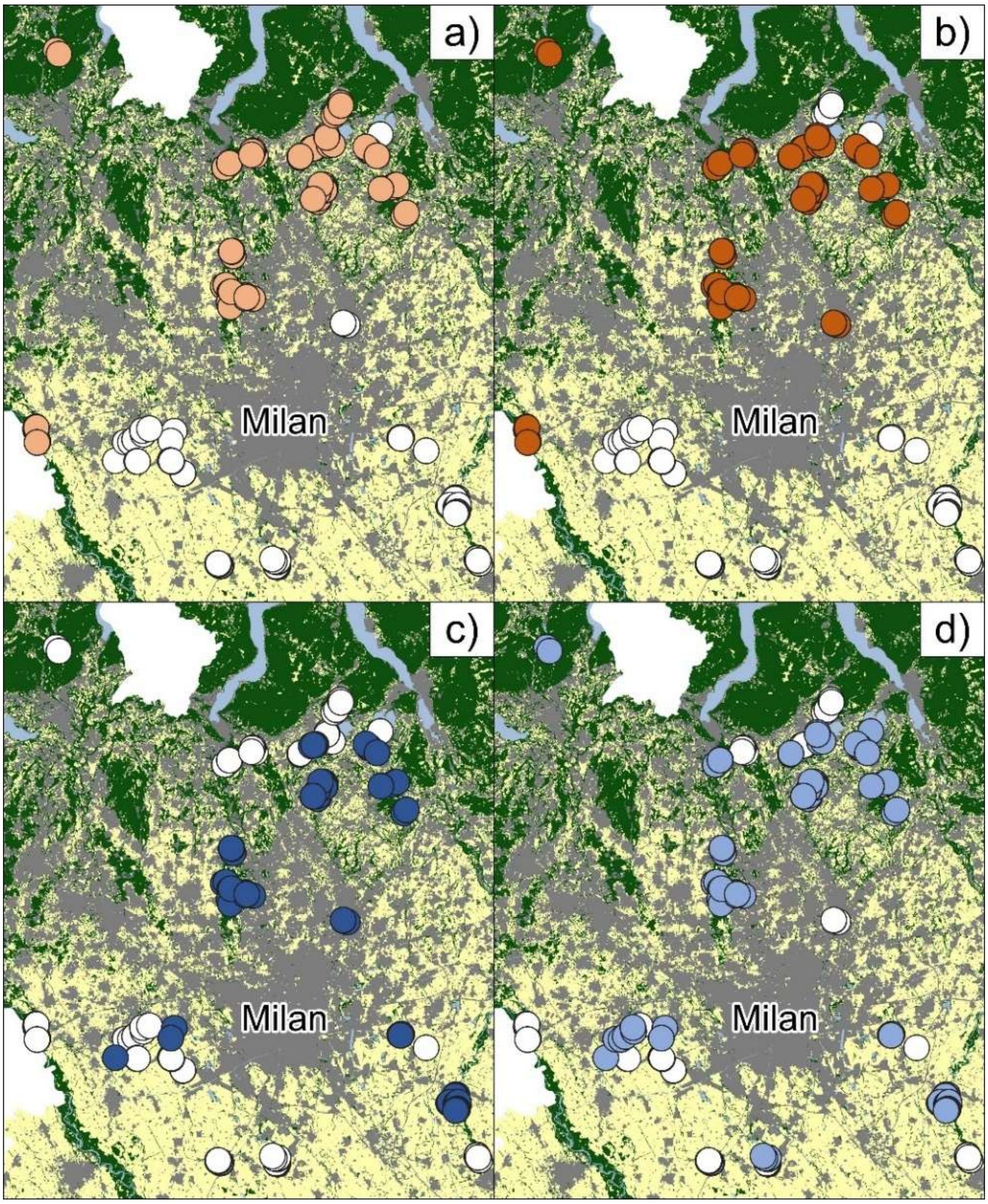

Appendix S3. Calculation of the mean occupancy and abundance across three discrete periods.

For each species and model (occupancy and abundance), we aggregated the data over three periods as follows:

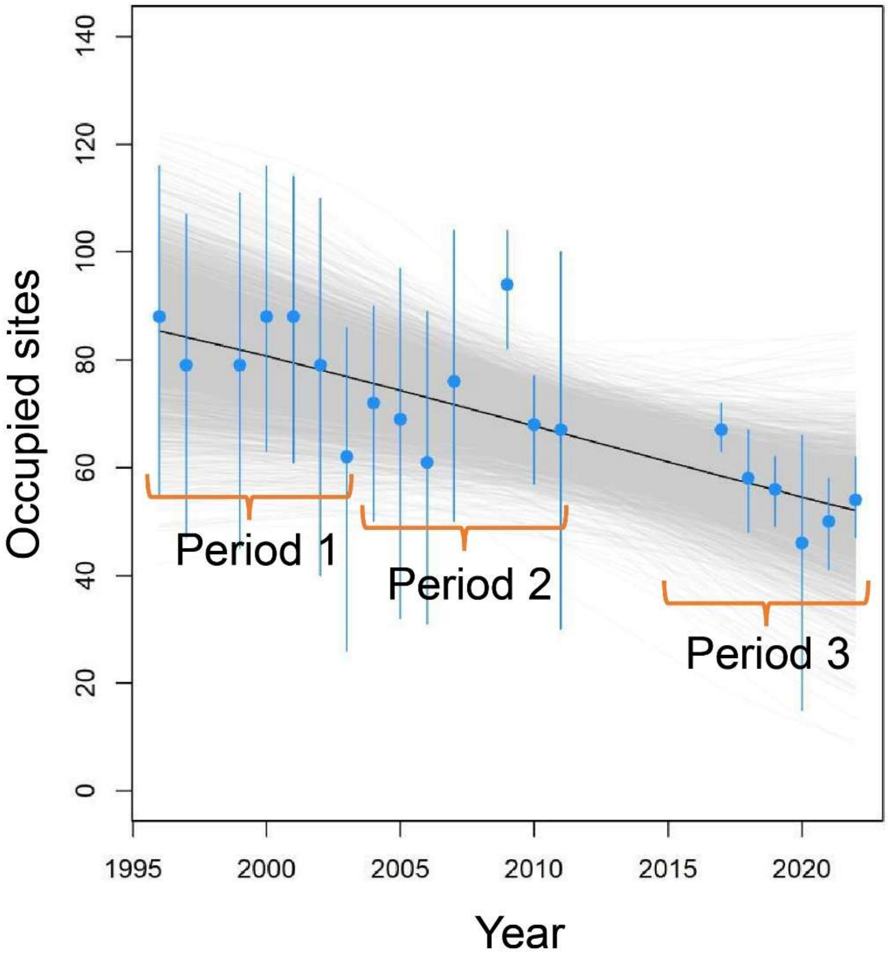

The figure above shows the trend of occupancy for *Rana latastei*, with blue bars representing estimates of total number of occupied sites in each year of sampling (dots: median, bars 95 % credible intervals). We chose the three periods by balancing the number of years present in each period that were represented in our dataset. Period 1 included seven years: 1996, 1997, 1999, 2000, 2001, 2002, 2003; period 2 included seven years: 2004, 2005, 2006, 2007, 2009, 2010, 2011; period 3 included the last six years of sampling: 2017, 2018, 2019, 2020, 2021, 2022. Within each period, we calculated derived parameters representing the mean estimates of total number of occupied sites and total abundance.

Appendix S4. Correction factors used to transform declines among periods to annual declines.

Species	Generation length (years)	3 × generation length (or 10 years)	Reference
Rana dalmatina	4	12	(Guarino et al. 1995)
Rana latastei	2.5	10	(Guarino et al. 2003)
Triturus carnifex	4.4	13.2	(Pagano et al. 1990)
Lissotriton vulgaris	3.35	10	(Nobili & Accordi 1997)

Appendix S5. Parameters estimated by occupancy and abundance models for the four study species. For each parameter, the mean estimate, standard deviation, median, 95% credible interval and Rhat are reported. alpha: intercept of occupancy or abundance; beta.trend: coefficient of the relationship between time and occupancy or abundance; prop.change: proportional population change between the last and the first year; sd.lpsi: standard deviation of the year random effect; sd.S: standard deviation of the site random effect; p: coefficients of the detection model (p_int: intercept, sd.p.S: standard deviation of the site random effect for the detection submodel, p_day and p_day2: linear and quadratic effect of the date of the survey, p_hour and p_hour2: linear and quadratic effects of the hour of the survey); occ[1 to 20]: total number of occupied sites in each year of sampling; Ntot[1 to 20]: total abundance across all sites in each year of sampling; v = dispersion parameter of the negative binomial distribution. a-d: occupancy models, e-h: abundance models presented in the main text, i-j: abundance models for newts without detection probability (not presented in the main text).

Parameter	Mean estimate	Standard deviation	Median estimate	95 % Credible Interval		Rhat
				Lower	Upper	
a) *Rana dalmatina* - Occupancy						
alpha	2.13	0.72	2.05	0.95	3.84	1.01
beta.trend	-0.01	0.07	-0.01	-0.16	0.14	1.01
prop.change	1.02	0.28	0.98	0.59	1.70	1.01
sd.lpsi	1.73	0.74	1.60	0.67	3.59	1.01
sd.S	2.87	0.56	2.80	1.92	4.12	1.02
p_int	-0.33	0.22	-0.32	-0.81	0.08	1.03
sd.p.S	1.73	0.18	1.72	1.41	2.12	1.01
p_day1	-0.87	0.07	-0.87	-1.00	-0.75	1.00
p_day2	0.15	0.05	0.15	0.04	0.25	1.00
p_hour1	-0.21	0.07	-0.21	-0.35	-0.08	1.00
p_hour2	0.10	0.06	0.10	0.00	0.22	1.00
occ[1]	96.26	18.91	99.00	52.00	124.00	1.00
occ[2]	82.50	15.18	83.00	49.98	110.00	1.00
occ[3]	91.00	20.02	93.00	46.00	123.00	1.00
occ[4]	90.01	23.26	94.00	33.00	123.00	1.00
occ[5]	89.98	22.82	94.00	35.98	123.00	1.00
occ[6]	89.72	22.84	94.00	35.00	123.00	1.00
occ[7]	95.40	14.69	96.00	65.00	122.00	1.00
occ[8]	101.89	11.39	102.00	79.00	123.00	1.00
occ[9]	76.59	20.16	78.00	36.00	112.00	1.00
occ[10]	67.95	18.46	69.00	31.00	102.00	1.00
occ[11]	102.83	12.91	104.00	75.00	124.00	1.00
occ[12]	96.18	6.49	96.00	84.00	109.00	1.01
occ[13]	116.77	5.45	117.00	104.00	125.00	1.01
occ[14]	89.58	21.72	93.00	36.98	122.00	1.00
occ[15]	91.31	4.60	91.00	83.00	101.00	1.01
occ[16]	104.68	6.13	105.00	93.00	116.00	1.01
occ[17]	82.62	4.88	83.00	73.00	92.00	1.01
occ[18]	101.39	12.09	102.00	77.00	123.00	1.00
occ[19]	90.40	6.18	90.00	79.00	103.00	1.01
occ[20]	75.99	6.39	76.00	64.00	89.00	1.00

Parameter	Mean estimate	Standard deviation	Median estimate	95 % Credible Interval Lower	Upper	Rhat
b) *Rana latastei* - Occupancy						
alpha	2.13	1.30	2.03	-0.58	5.55	1.09
beta.trend	-0.19	0.13	-0.18	-0.51	0.01	1.01
prop.change	0.62	0.20	0.62	0.20	1.02	1.01
sd.lpsi	2.63	1.56	2.26	0.85	7.63	1.05
sd.S	5.20	1.45	4.97	2.99	8.56	1.01
p_int	-0.90	0.20	-0.89	-1.30	-0.52	1.02
sd.p.S	1.28	0.18	1.27	0.95	1.67	1.00
p_day1	-0.46	0.06	-0.46	-0.59	-0.34	1.00
p_day2	-0.01	0.04	-0.01	-0.09	0.07	1.00
p_hour1	0.16	0.07	0.15	0.01	0.30	1.00
p_hour2	0.06	0.06	0.06	-0.04	0.18	1.01
occ[1]	101.32	16.56	104.00	64.00	126.00	1.00
occ[2]	96.55	17.88	99.00	57.00	125.00	1.01
occ[3]	94.61	19.56	97.00	51.00	125.00	1.00
occ[4]	103.95	12.67	105.00	78.00	126.00	1.00
occ[5]	103.39	12.54	104.00	78.00	125.00	1.01
occ[6]	91.22	21.24	94.00	40.00	124.00	1.00
occ[7]	70.71	20.48	74.00	19.00	100.03	1.00
occ[8]	86.98	11.59	88.00	62.00	108.00	1.00
occ[9]	78.29	20.53	82.00	27.00	110.00	1.00
occ[10]	75.63	18.87	75.00	40.00	114.00	1.00
occ[11]	94.43	13.73	95.00	67.00	121.00	1.01
occ[12]	113.78	6.24	114.00	101.00	126.00	1.02
occ[13]	84.49	7.37	85.00	70.00	99.00	1.00
occ[14]	78.76	23.21	82.00	22.00	118.00	1.01
occ[15]	83.33	5.38	83.00	73.00	94.00	1.02
occ[16]	73.79	7.41	74.00	60.00	89.00	1.01
occ[17]	68.59	5.80	68.00	57.00	81.00	1.01
occ[18]	52.45	19.71	55.00	6.98	85.00	1.01
occ[19]	60.03	6.93	60.00	47.00	75.00	1.00
occ[20]	65.38	5.51	65.00	55.00	77.00	1.00
c) *Triturus carnifex* - Occupancy						
alpha	0.66	1.31	0.58	-1.72	3.59	1.01
beta.trend	-0.34	0.19	-0.32	-0.74	-0.01	1.02
prop.change	0.47	0.21	0.43	0.15	0.98	1.02
sd.lpsi	4.38	1.92	4.20	1.10	8.82	1.00
sd.S	7.40	1.65	7.54	4.03	9.89	1.00
p_int	-1.61	0.31	-1.58	-2.29	-1.05	1.00
sd.p.S	1.23	0.27	1.21	0.80	1.83	1.01
p_day1	0.71	0.09	0.71	0.52	0.89	1.00
p_day2	-0.23	0.05	-0.23	-0.35	-0.13	1.00
p_hour1	0.35	0.10	0.35	0.17	0.55	1.00
p_hour2	0.16	0.07	0.16	0.02	0.31	1.00
occ[1]	78.37	22.77	82.00	25.00	110.00	1.01
occ[2]	88.41	15.03	90.00	55.00	111.00	1.00
occ[3]	87.01	15.64	89.00	53.00	110.00	1.00
occ[4]	85.21	15.09	86.00	53.00	109.03	1.00
occ[5]	76.42	17.69	77.00	41.00	108.00	1.00
occ[6]	81.96	11.42	81.00	61.00	106.00	1.00
occ[7]	41.24	16.31	36.00	22.00	86.00	1.03
occ[8]	36.24	17.29	32.00	15.00	83.00	1.03
occ[9]	64.17	23.32	65.00	19.00	106.03	1.00

Parameter	Mean estimate	Standard deviation	Median estimate	95 % Credible Interval Lower	Upper	Rhat
occ[10]	64.16	23.69	65.00	14.00	106.00	1.00
occ[11]	62.24	23.10	63.00	15.00	105.00	1.00
occ[12]	48.33	12.69	47.00	28.00	77.00	1.00
occ[13]	53.64	9.91	53.00	38.00	76.00	1.01
occ[14]	83.68	15.18	85.00	54.00	109.00	1.00
occ[15]	44.53	5.88	44.00	35.00	58.00	1.00
occ[16]	53.50	6.39	53.00	43.00	68.00	1.00
occ[17]	34.74	7.09	34.00	22.00	50.00	1.00
occ[18]	64.24	14.91	63.00	37.00	96.00	1.00
occ[19]	36.32	5.70	36.00	27.00	49.00	1.00
occ[20]	26.71	5.77	26.00	16.00	39.00	1.00
d) *Lissotriton vulgaris* - Occupancy						
alpha	-0.69	1.33	-0.69	-3.31	1.93	1.00
beta.trend	-0.25	0.20	-0.23	-0.68	0.10	1.00
prop.change	0.57	0.30	0.51	0.18	1.30	1.00
sd.lpsi	4.59	1.82	4.34	1.74	8.86	1.00
sd.S	7.86	1.55	8.11	4.47	9.94	1.00
p_int	-1.45	0.46	-1.37	-2.58	-0.76	1.01
sd.p.S	1.26	0.35	1.21	0.71	2.06	1.01
p_day1	0.23	0.08	0.22	0.08	0.38	1.00
p_day2	-0.10	0.07	-0.10	-0.24	0.03	1.00
p_hour1	0.28	0.10	0.28	0.10	0.47	1.00
p_hour2	0.18	0.07	0.18	0.04	0.32	1.00
occ[1]	72.10	27.57	72.00	19.00	123.03	1.00
occ[2]	90.40	21.16	91.00	48.98	127.00	1.00
occ[3]	90.14	20.42	90.00	52.00	127.00	1.00
occ[4]	72.22	27.79	73.00	19.00	124.00	1.00
occ[5]	70.85	27.73	71.00	17.00	123.00	1.00
occ[6]	83.58	15.23	82.00	59.00	118.00	1.00
occ[7]	32.09	10.35	30.00	18.00	55.00	1.01
occ[8]	47.76	29.14	35.00	16.00	114.00	1.01
occ[9]	58.17	28.30	55.00	14.00	116.00	1.00
occ[10]	45.69	21.12	44.00	8.00	91.03	1.00
occ[11]	65.78	20.12	65.00	28.00	108.00	1.00
occ[12]	43.43	11.49	41.00	27.00	72.00	1.00
occ[13]	68.17	16.14	66.00	43.98	106.00	1.00
occ[14]	90.43	17.45	90.00	58.00	124.00	1.00
occ[15]	39.43	9.05	38.00	26.00	61.00	1.01
occ[16]	51.37	8.72	50.00	39.00	73.00	1.01
occ[17]	40.29	9.14	39.00	27.00	62.03	1.01
occ[18]	60.61	15.52	58.00	37.00	99.00	1.00
occ[19]	47.16	9.62	45.00	33.98	71.00	1.01
occ[20]	21.50	7.22	21.00	10.00	38.00	1.00
e) *Rana dalmatina* - Abundance						
alpha	0.82	0.39	0.84	-0.03	1.55	1.07
beta.trend	-0.02	0.03	-0.02	-0.07	0.04	1.03
prop.change	0.80	0.76	0.60	0.14	2.89	1.03
sd.lpsi	0.58	0.41	0.46	0.16	1.82	1.03
sd.S	3.00	0.28	2.98	2.51	3.59	1.00
v	0.65	0.06	0.65	0.54	0.79	1.01
Ntot[1]	13134.29	25766.03	7638.00	1954.93	56544.28	1.01
Ntot[2]	6555.62	4948.81	5343.00	1563.83	18898.98	1.01

Parameter	Mean estimate	Standard deviation	Median estimate	95 % Credible Interval Lower	Upper	Rhat
Ntot[3]	6273.12	6576.17	4853.50	1148.93	19634.80	1.02
Ntot[4]	6866.88	12155.29	4612.00	653.88	23604.65	1.04
Ntot[5]	6768.42	13465.29	4648.00	642.88	21769.15	1.04
Ntot[6]	7091.55	34921.66	4538.50	712.78	22742.35	1.21
Ntot[7]	3540.29	2112.61	3034.00	1067.98	9051.03	1.03
Ntot[8]	3473.98	3112.77	3025.50	1257.98	8039.25	1.01
Ntot[9]	2569.58	2201.11	2202.00	27.98	7673.28	1.02
Ntot[10]	3503.91	2430.64	3058.00	427.90	9349.83	1.01
Ntot[11]	3327.59	2359.75	2876.00	423.00	9018.50	1.02
Ntot[12]	4141.75	1996.69	3821.50	3129.00	6782.28	1.02
Ntot[13]	8952.31	2387.62	8421.00	7118.93	13576.00	1.01
Ntot[14]	5561.11	22282.86	3757.50	677.93	16479.18	1.13
Ntot[15]	2460.70	257.57	2398.00	2288.00	3042.10	1.02
Ntot[16]	2446.98	697.75	2271.00	2038.00	3845.28	1.04
Ntot[17]	3595.31	744.78	3447.00	3011.98	4943.08	1.00
Ntot[18]	3249.37	1302.07	2945.50	1796.98	6518.10	1.01
Ntot[19]	3531.87	793.43	3312.00	3028.00	5318.38	1.01
Ntot[20]	2913.10	1136.31	2680.50	1559.90	5597.28	1.00
f) *Rana latastei* - Abundance						
alpha	-0.53	0.52	-0.52	-1.57	0.46	1.04
beta.trend	-0.08	0.05	-0.08	-0.19	0.02	1.00
prop.change	0.53	3.20	0.11	0.01	1.84	1.00
sd.lpsi	1.28	0.53	1.19	0.53	2.59	1.05
sd.S	2.82	0.34	2.80	2.22	3.57	1.01
v	0.21	0.03	0.21	0.15	0.28	1.00
Ntot[1]	30541.11	345847.42	5838.50	424.90	150713.23	1.28
Ntot[2]	33730.49	128303.40	10635.50	1111.68	189066.28	1.08
Ntot[3]	9013.06	48040.36	2041.50	68.00	54952.33	1.02
Ntot[4]	7209.15	20364.23	2422.50	210.00	40437.45	1.00
Ntot[5]	5102.93	29747.36	1723.50	323.00	25191.15	1.00
Ntot[6]	19985.59	299849.40	2155.50	78.98	75329.03	1.25
Ntot[7]	2060.70	7749.39	735.00	23.00	10713.68	1.04
Ntot[8]	4494.55	14972.20	2030.50	280.00	21577.45	1.10
Ntot[9]	3385.64	13207.13	1024.00	32.00	18530.13	1.03
Ntot[10]	1842.90	6463.11	627.50	19.00	9907.78	1.10
Ntot[11]	5396.98	18250.33	1857.00	175.00	31527.18	1.10
Ntot[12]	5304.34	12159.56	2707.50	609.00	24307.65	1.12
Ntot[13]	592.53	1131.77	336.00	127.00	2514.30	1.02
Ntot[14]	5589.40	44681.74	1053.00	41.98	21512.38	1.25
Ntot[15]	1284.24	1679.21	938.00	786.00	3634.18	1.04
Ntot[16]	355.36	538.57	233.00	131.00	1284.05	1.01
Ntot[17]	740.81	1115.32	468.00	217.98	2756.18	1.03
Ntot[18]	485.46	1424.72	190.50	6.00	2729.10	1.09
Ntot[19]	634.96	499.32	514.00	401.00	1494.25	1.01
Ntot[20]	1126.02	2306.03	735.00	494.00	3708.55	1.01
g) *Triturus carnifex* - Abundance (with detection probability)						
alpha	-1.81	0.55	-1.80	-2.93	-0.80	1.00
beta.trend	-0.08	0.05	-0.08	-0.17	0.01	1.01
prop.change	0.26	0.66	0.12	0.01	1.47	1.01
sd.lpsi	1.27	0.39	1.21	0.71	2.22	1.02
sd.S	2.47	0.34	2.43	1.90	3.21	1.01
v	0.65	0.15	0.63	0.40	0.99	1.00

Parameter	Mean estimate	Standard deviation	Median estimate	95 % Credible Interval Lower	Upper	Rhat
p	0.79	0.15	0.82	0.40	0.99	1.01
Ntot[1]	2823.41	24698.24	557.50	21.00	14074.63	1.28
Ntot[2]	1217.33	2301.79	553.00	47.00	6471.35	1.08
Ntot[3]	879.59	1598.27	420.00	38.98	4706.68	1.04
Ntot[4]	2197.29	3561.57	1151.50	174.00	11289.13	1.01
Ntot[5]	1095.74	1827.24	570.00	87.00	5290.88	1.03
Ntot[6]	2867.07	3042.39	2027.00	474.98	10580.28	1.00
Ntot[7]	221.59	172.79	172.00	74.98	686.00	1.05
Ntot[8]	121.46	130.68	85.00	22.00	447.03	1.01
Ntot[9]	225.90	443.72	111.00	6.00	1131.08	1.03
Ntot[10]	970.66	6336.44	253.50	11.00	4745.05	1.02
Ntot[11]	920.66	4963.17	227.50	9.00	5091.20	1.19
Ntot[12]	275.91	127.56	241.00	155.00	622.05	1.02
Ntot[13]	31.49	28.31	24.00	9.00	102.00	1.00
Ntot[14]	263.55	585.86	116.00	6.00	1449.20	1.04
Ntot[15]	222.93	78.21	201.00	154.00	430.05	1.00
Ntot[16]	403.66	275.67	328.00	187.00	1059.00	1.02
Ntot[17]	110.44	57.11	95.00	60.00	256.03	1.01
Ntot[18]	75.43	78.70	54.00	14.00	268.00	1.01
Ntot[19]	83.83	26.86	76.00	59.00	158.00	1.03
Ntot[20]	44.43	17.68	40.00	26.00	94.00	1.02
h) *Lissotriton vulgaris* - Abundance (with detection probability)						
alpha	-1.46	0.52	-1.43	-2.49	-0.51	1.05
beta.trend	-0.09	0.03	-0.09	-0.15	-0.02	1.03
prop.change	0.15	0.19	0.10	0.02	0.65	0.15
sd.lpsi	0.78	0.31	0.74	0.29	1.52	1.02
sd.S	2.57	0.35	2.54	1.96	3.33	1.01
v	0.37	0.06	0.37	0.27	0.50	1.00
p	0.76	0.16	0.77	0.45	0.99	1.10
Ntot[1]	1113.77	1420.40	679.50	76.00	4747.75	1.02
Ntot[2]	1040.96	1334.47	658.50	96.98	4255.45	1.05
Ntot[3]	2127.65	3179.21	1258.00	282.98	8767.48	1.02
Ntot[4]	1273.93	3487.69	663.00	81.00	5651.15	1.12
Ntot[5]	1104.41	2358.49	616.00	73.98	5176.08	1.03
Ntot[6]	2059.53	1856.89	1495.50	466.00	7094.03	1.00
Ntot[7]	690.60	553.32	540.50	224.98	2038.35	1.02
Ntot[8]	286.02	268.72	213.00	56.00	976.08	1.04
Ntot[9]	401.82	505.55	263.50	24.00	1589.15	1.05
Ntot[10]	451.76	538.10	296.00	35.98	1796.05	1.01
Ntot[11]	566.11	674.75	379.50	57.98	2152.00	1.01
Ntot[12]	372.55	259.48	307.00	161.00	960.23	1.02
Ntot[13]	152.59	105.23	123.50	55.00	432.03	1.04
Ntot[14]	403.92	1340.16	229.50	36.00	1653.28	1.01
Ntot[15]	111.76	43.29	101.00	69.00	213.03	1.08
Ntot[16]	236.43	151.41	206.00	139.00	485.03	1.05
Ntot[17]	292.14	114.14	265.50	174.00	565.10	1.03
Ntot[18]	173.93	166.88	137.00	54.00	496.28	1.05
Ntot[19]	112.30	60.09	98.00	65.00	224.05	1.08
Ntot[20]	53.67	37.07	43.00	19.00	144.00	1.03
i) *Triturus carnifex* - Abundance (without detection probability)						
alpha	-2.02	0.49	-2.01	-2.98	-1.10	1.04
beta.trend	-0.09	0.05	-0.09	-0.18	0.00	1.01

Parameter	Mean estimate	Standard deviation	Median estimate	95 % Credible Interval		Rhat
				Lower	Upper	
prop.change	0.23	0.44	0.11	0.01	1.13	1.01
sd.lpsi	1.24	0.36	1.18	0.70	2.11	1.01
sd.S	2.48	0.35	2.45	1.87	3.21	1.04
v	0.65	0.15	0.63	0.42	0.98	1.00
Ntot[1]	1909.92	8575.63	480.50	23.98	11052.55	1.06
Ntot[2]	1026.95	2510.66	469.00	42.00	5470.43	1.02
Ntot[3]	729.95	1481.22	346.00	32.00	3512.85	1.02
Ntot[4]	1722.49	3613.08	879.50	149.00	8541.30	1.07
Ntot[5]	818.51	1317.80	435.50	73.00	4218.60	1.03
Ntot[6]	2114.81	1933.23	1569.00	408.98	6915.33	1.02
Ntot[7]	168.88	124.20	133.00	64.00	478.08	1.01
Ntot[8]	90.69	81.42	66.00	19.00	315.03	1.02
Ntot[9]	173.06	294.59	91.00	5.00	834.23	1.03
Ntot[10]	868.96	8766.48	196.00	12.00	4009.10	1.26
Ntot[11]	739.46	4507.96	196.00	10.00	3694.83	1.09
Ntot[12]	209.52	77.77	187.00	143.00	415.03	1.02
Ntot[13]	23.05	18.51	18.00	7.00	70.00	1.00
Ntot[14]	216.11	1062.99	89.00	6.00	1067.15	1.07
Ntot[15]	166.62	28.02	159.00	146.00	233.00	1.04
Ntot[16]	300.83	199.55	252.00	163.00	715.05	1.01
Ntot[17]	82.09	33.65	73.00	55.00	173.00	1.03
Ntot[18]	54.79	51.93	40.00	11.00	192.00	1.03
Ntot[19]	62.49	6.50	61.00	56.00	79.00	1.02
Ntot[20]	33.26	8.61	31.00	23.00	55.00	1.01
j) *Lissotriton vulgaris* - Abundance (without detection probability)						
alpha	-1.80	0.48	-1.78	-2.88	-0.90	1.05
beta.trend	-0.09	0.04	-0.09	-0.15	-0.02	1.01
prop.change	0.18	0.40	0.11	0.02	0.64	1.01
sd.lpsi	0.82	0.36	0.75	0.29	1.73	1.00
sd.S	2.61	0.37	2.57	2.01	3.40	1.04
v	0.37	0.06	0.37	0.27	0.50	1.01
Ntot[1]	858.32	1306.21	513.50	45.00	3721.18	1.06
Ntot[2]	786.06	991.93	504.00	68.00	3542.35	1.01
Ntot[3]	1683.80	2930.17	955.00	205.00	7689.03	1.07
Ntot[4]	1065.13	3363.95	515.50	48.00	4792.55	1.08
Ntot[5]	817.07	1449.66	449.00	47.00	3685.08	1.06
Ntot[6]	1625.85	1591.20	1172.50	397.98	5554.58	1.01
Ntot[7]	513.44	385.18	402.00	183.00	1552.18	1.01
Ntot[8]	217.05	227.99	162.00	46.00	688.10	1.05
Ntot[9]	292.05	385.77	192.00	15.00	1154.13	1.04
Ntot[10]	329.85	403.47	226.50	21.00	1195.23	1.04
Ntot[11]	439.62	804.50	277.50	43.98	1545.58	1.05
Ntot[12]	271.72	162.59	229.00	143.00	633.05	1.03
Ntot[13]	114.20	85.79	93.00	47.00	316.05	1.00
Ntot[14]	266.22	389.07	171.00	23.00	1010.05	1.01
Ntot[15]	80.67	23.05	75.00	64.00	129.00	1.02
Ntot[16]	173.01	87.26	153.00	130.00	332.03	1.10
Ntot[17]	213.19	63.13	197.00	162.00	379.08	1.02
Ntot[18]	129.67	100.32	104.00	46.00	358.08	1.05
Ntot[19]	82.34	30.24	74.00	60.00	155.08	1.02
Ntot[20]	39.14	25.36	32.00	16.00	101.00	1.02

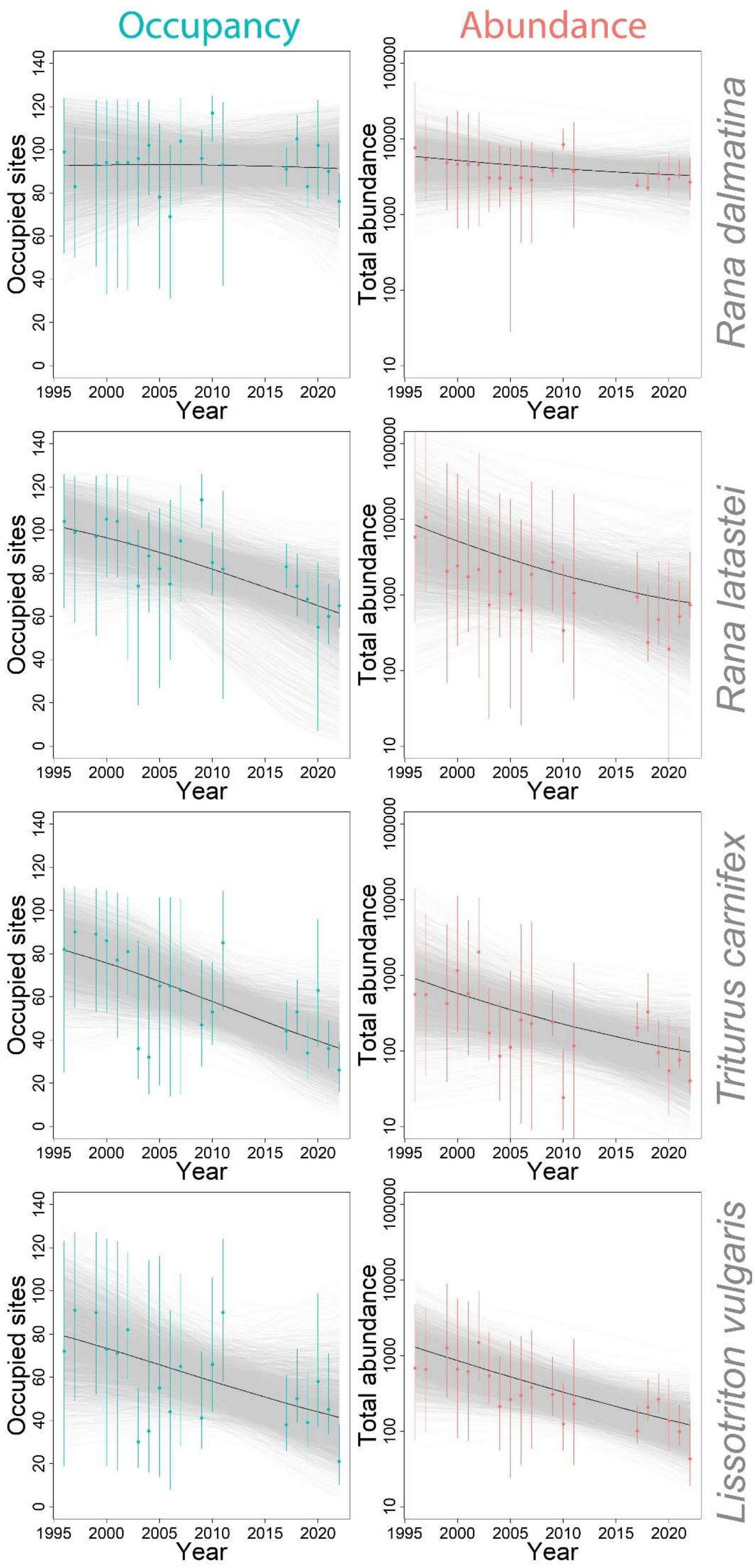

Appendix S6. Species-specific trends of occupancy and abundance. Plots show the total number of occupied sites estimated by occupancy models (azure) and the total abundance estimated by abundance models (red), in each year of sampling. Dots represent the median occupancy and abundance, and bars represent the 95 % credible intervals. In each plot, the black line represents the average trend, while grey lines are realisations from 3,000 posteriors for the trend parameter (β_{trend}). Estimates of occupancy and abundance for years where no surveys were performed are not shown.

Appendix S7. Comparison of the results of abundance models for *Triturus carnifex* and *Lissotriton vulgaris*, with or without including a detection component in the models.

Models with or without a detection component estimated very similar abundance values and overlapping abundance trends, as shown in the figure below:

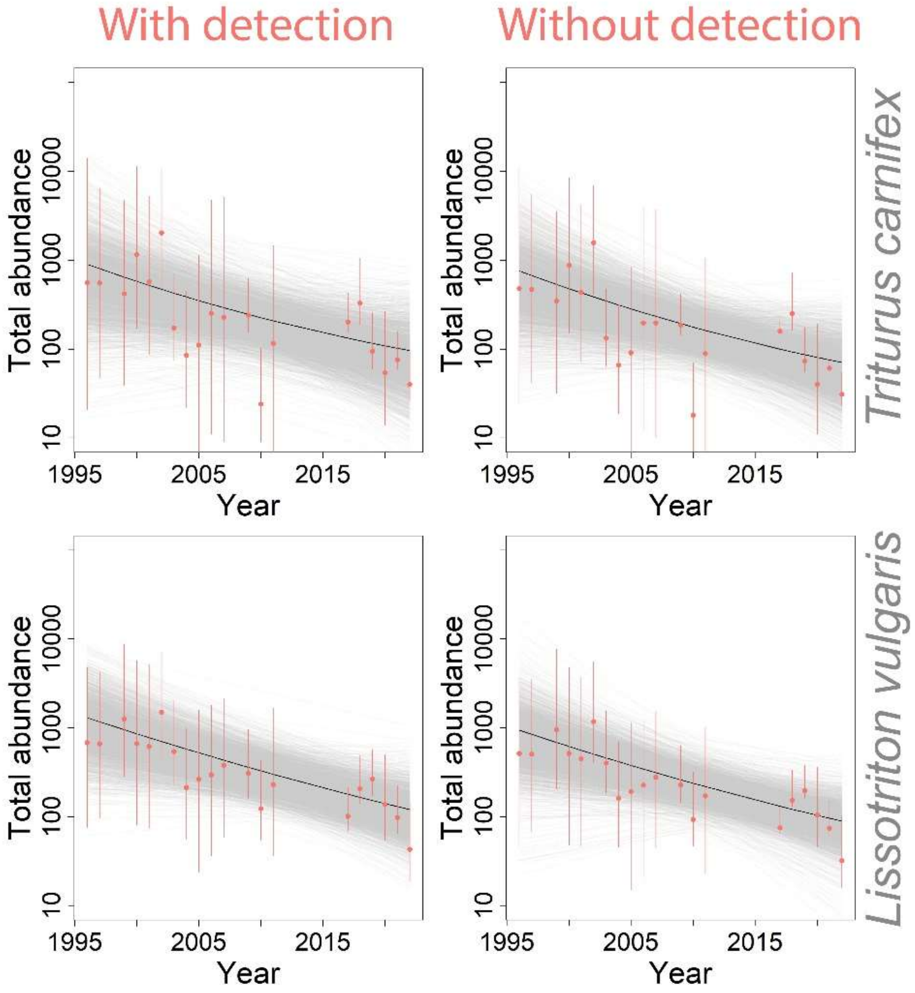

Additionally, decline rates and decline probabilities estimated by the two alternative model types were largely overlapping, as shown in the table below:

		With detection probability			Without detection probability		
			Decline rate			Decline rate	
Species	Period	Decline probability	Median	95% Credible interval	Decline probability	Median	95% Credible interval
Triturus carnifex	$\tau_1 - \tau_2$	0.97	-0.92	-0.99 / +0.28	0.97	-0.92	-0.99 / +0.14
	$\tau_2 - \tau_3$	0.75	-0.36	-0.92 / +1.13	0.75	-0.37	-0.92 / +1.11
Lissotriton vulgaris	$\tau_1 - \tau_2$	0.99	-0.77	-0.95 / -0.25	0.99	-0.78	-0.96 / -0.27
	$\tau_2 - \tau_3$	0.96	-0.45	-0.75 / +0.08	0.96	-0.45	-0.75 / +0.10

References

Ficetola GF, De Bernardi F. 2004. Amphibians in a human-dominated landscape: The community structure is related to habitat features and isolation. Biological Conservation **119**:219–230.

Godsoe W. 2010. I can't define the niche but i know it when i see it: A formal link between statistical theory and the ecological niche. Oikos **119**:53–60.

Guarino FM, Angelini F, Cammarota M. 1995. A skeletochronological analysis of three syntopic amphibian species from southern Italy. Amphibia-Reptilia **16**:297–302.

Guarino FM, Lunardi S, Carlomagno M, Mazzotti S. 2003. A skeletochronological study of growth, longevity, and age at sexual maturity in a population of *Rana latastei* (Amphibia, Anura). Journal of Biosciences **28**:775–782.

Jeliazkov A, Chiron F, Garnier J, Besnard A, Silvestre M, Jiguet F. 2014. Level-dependence of the relationships between amphibian biodiversity and environment in pond systems within an intensive agricultural landscape. Hydrobiologia **723**:7–23.

Nobili G, Accordi F. 1997. Body size, age and fecundity variation in different populations of the smooth newt triturus vulgaris meridionalis in central Italy. Italian Journal of Zoology **64**:313–318.

Pagano M, Rossi F, Cavallotto L, Campolongo P, Giacoma C. 1990. Age determination in *Triturus carnifex*. Atti VI Convegno Nazionale Associazione "Alessandro Ghigi" (Torino, 22-24 June 1989). Museo Regionale di Scienze Naturali di Torino, Torino, Italy.

Smith MA, Green DM. 2005. Dispersal and the metapopulation paradigm in amphibian ecology and conservation: are all amphibian populations metapopulations? Ecography **28**:110–128.
